# Amino Acid-Derived Quorum Sensing Molecule Alanine on the Gastrointestinal Tract Tolerance of the *Lactobacillus* Strains in the Cocultured Fermentation Model

**DOI:** 10.1128/spectrum.00832-21

**Published:** 2022-03-03

**Authors:** Jianzhu Wen, Yixing Yu, Mengqi Chen, Lei Cui, Qiang Xia, Xiaoqun Zeng, Yuxing Guo, Daodong Pan, Zhen Wu

**Affiliations:** a State Key Laboratory for Managing Biotic and Chemical Threats to the Quality and Safety of Agro-products, Ningbo University, Ningbo, Zhejiang, China; b Key Laboratory of Animal Protein Deep Processing Technology of Zhejiang, School of Food and Pharmaceutical Sciences, Ningbo University, Ningbo, Zhejiang, China; c School of Food Science & Pharmaceutical Engineering, Nanjing Normal University, Nanjing, People’s Republic of China; University of Thessaly

**Keywords:** GIT tolerance, amino acid metabolism, coculture condition, *Lactobacillus*, quorum sensing

## Abstract

More and more people are aware of the importance of intestinal flora to human health, and people are interested in the regulation of intestinal flora and its interaction with the host. The survival status of the probiotics in the gastrointestinal environment and the microbial interactions between the lactic acid bacteria have also received considerable attention. In this study, the gastrointestinal environment tolerance, adhesion ability, and biofilm formation of the *Lactobacillus* strain in the coculture system were explored through the real-time fluorescence-based quantitative PCR, UPLC-MS/MS metabolic profiling analysis, and Live/Dead BacLight cell staining methods. The results showed that the coculture system could promote the release of signal molecules autoinducer-2 and effectively protect the viability of the Lactobacillus acidophilus in the gastrointestinal environment. Meanwhile, amino acid-derived characteristic metabolite l-alanine (1%) could effectively enhance the communication of the cells in the complex fermentation model, which led to an increase in the tolerance ability of the L. acidophilus by 28% in the gastrointestinal-like environment.

**IMPORTANCE** It was deduced from the study that amino acid-derived metabolites play an important role in cell communication in the gastrointestinal tract (GIT) environment, thus enhancing the communication of *Lactobacillus* strains in the complex fermentation model. Meanwhile, the viability of Lactobacillus acidophilus can be increased in the coculture system during the gastrointestinal stress environment treated with the amino acid-derived quorum sensing (QS) molecule l-alanine. It will shed some light on the application of amino acid-derived QS molecules in the fermentation stater industry.

## INTRODUCTION

Probiotics are defined as living organisms when administered in adequate amounts confer a health benefit on the host (1, 2). The microorganisms most used as probiotics include *Lactobacillus*, *Bifidobacterium*, and *Saccharomyces*. Other strains, including *Bacillus*, *Propionibacterium*, Streptococcus, and Escherichia, are also considered novel probiotics (3). They are thought to mainly exert their beneficial effects by modulating the endocrine system, central nervous system (CNS), skin, and urogenital tract (via local and systemic immuno-modulation). In addition, as one of the widely used lactic acid bacteria (LAB), *Lactobacillus* is commonly used in the fermentation food industry in the coculture fermentation models with some eximious probiotics, which can improve the nutritional value of the fermented foods (4, 5). However, the interaction mechanism between different strains in this matrix remains unclear.

It is believed that a special interaction mode exists in bacteria communication, and extracellular signaling molecules in quorum sensing are a common way in this mode (6, 7). In addition, there is also a regulatory mechanism in this communication process between bacterial groups and many critical physiological functions, such as bioluminescence, motility, production of secondary metabolites, and biofilm formation (8, 9). Bacterial type, bacterial density, and environmental stimuli, including acid-base stress, and bacterial surface adhesion have been reported to affect the expression of quorum-sensing (QS)-related signaling molecules (10). In Gram-negative bacteria, N-acyl homoserine lactone is the most common signaling molecule (11). In addition, recent studies have found that the most common signaling molecules in Gram-positive bacteria are signaling peptides (autoinducing peptide [AIP]) and autoinducer-2 (AI-2) (12–14). AIP is transduced by a two-component condition of a transmembrane histidine kinase (HK) and transcription regulator (RR) (15). AgrD encodes the precursor of the AIP, whereas the integral membrane protein AgrB is involved in its processing and secretion of the thiolactone-modified cyclic oligopeptide. AI-2 is a signal molecule produced by LuxS, an enzyme found in many bacterial species, and, thus, proposed to enable interspecies communication (16).

AI-2 is considered to be a ‘universal’ signaling molecule for interspecies communication (8, 17). In the metabolic process, the use of S-(5′-adenosyl)-l-methionine (SAM) as a methyl donor produces the intermediate S-adenosylhomocysteine (SAH), which is hydrolyzed to S-ribosylhomocysteine (SRH) and adenine by the nucleosidase enzyme Pfs (5’methylthioadenosine/S-adenosylhomocysteine nucleosidase). LuxS catalyzes the cleavage of SRH to 4, 5-dihydroxy 2, 3-pentanedione (DPD), and homocysteine (18). Over the past few years, QS-related cell-to-cell communication in LAB has received more and more attention, especially in the coculture condition. Some researchers also found that QS affects the type and abundance of bacterial metabolites in the coculture condition (19). Meanwhile, whether the type and abundance of bacterial metabolites will also affect the synthesis of AIP and AI-2 is still uncertain and requires further investigation.

As previously reported, the main metabolites of LAB are amino acids, extracellular polysaccharides, and lactic acid (20). This study intended to explore the QS network of *Lactobacillus* in the coculture condition, the biofilm changes, metabolite profiles, as well as the liability of the *Lactobacillus* strains in the simulated gastrointestinal conditions were also investigated according to the amino metabolites-mediated QS changes. Our study improved understanding of the regulatory mechanism of QS on the tolerance behavior of the *Lactobacillus* strains in the gastrointestinal microenvironment and the influence of certain amino acids in the synthesis of AI-2 in the QS system.

## RESULTS

### Growth characteristics of the strains in coculture conditions.

**FIG 1:**
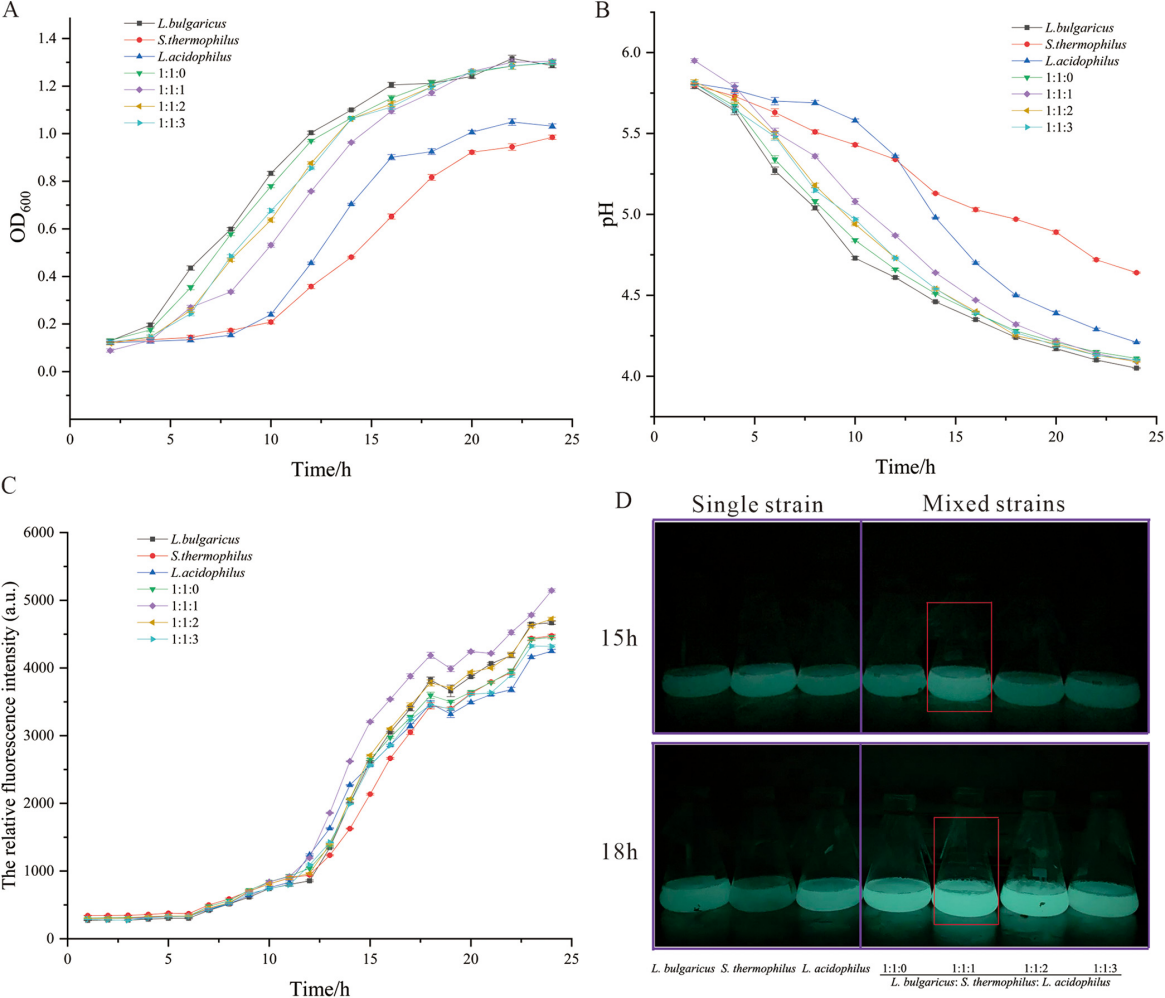
The screening process of the lactobacillus strains in the coculture condition. (A) The OD_600_ of cocultivation strains. (B) The pH of culture media during the culture condition. (C) The relative fluorescence intensity of AI-2 in the cocultivation condition. (D) The fluorescence image of the culture media in the single and mixed culture condition.

**FIG 2:**
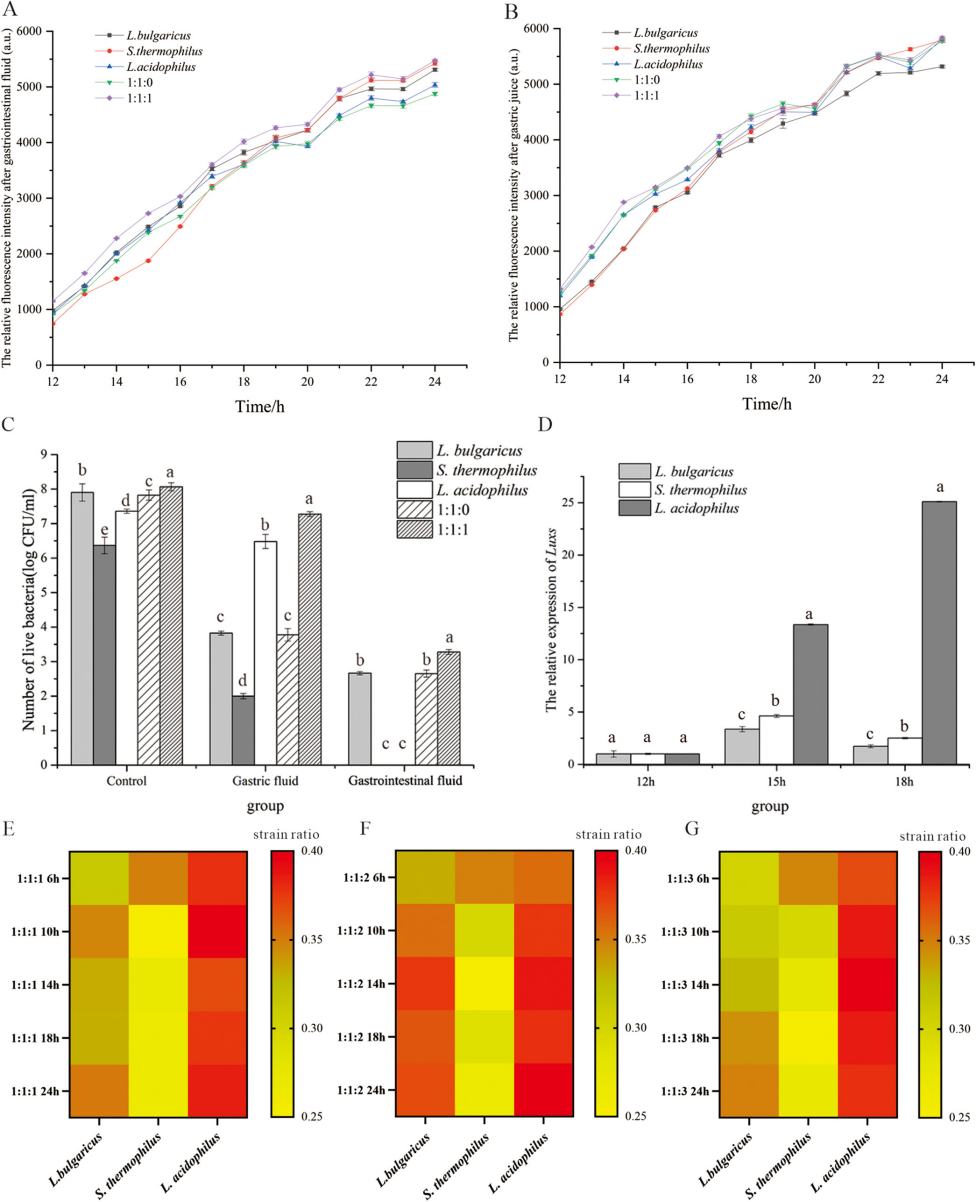
The influence of gastrointestinal condition on the growth characteristics of bacteria under coculture conditions. (A) The relative fluorescence intensity of the mixed strains in coculture condition after gastric juice treatment. (B) The relative fluorescence intensity of the mixed strains in coculture condition after gastrointestinal fluid treatment. (C) The number of live bacteria in the gastrointestinal fluid under coculture conditions. (D) The *LuxS* gene expression of the strains under the coculture conditions. (E) The ratio of single strain under the 1:1:1 coculture condition. (F) The ratio of the single strain under the 1:1:2 coculture condition. (G) The ratio of single strain in 1:1:3 coculture condition. Lowercase letters indicate differences within groups, *P* < 0.05 was used to indicate significant differences.

It can be observed from the optical density at 600 nm (OD_600_) that the bacterial density was lower when S. thermophilus and L. acidophilus were cultured separately, while the bacterial density was increased when the bacteria were cultured together. However, there was no significant difference between the changes of the mix strain ratio (*P* < 0.05 was considered a significant difference). In the mono-bacterial fermentation condition, L. bulgaricus had the highest OD value compared with L. acidophilus and S. thermophilus with an OD_600_ of 1.2 when cultured to 18 h while the growth rate of S. thermophilus was the lowest. The results showed that both the OD_600_ and pH at 24 h were more significant in the coculture condition than the condition of single fermentation of S. thermophilus (*P* = 0.0026 and 0.0047, respectively; Fig. 1A and B). When the inoculation ratios of L. bulgaricus, S. thermophilus, and L. acidophilus were 1:1:1, 1:1:2, or 1:1:3, the abundance of L. bulgaricus was decreased in the starter cultures while the quantity of L. acidophilus in the 1:1:3 group at 24 h was significantly increased compared to the 1:1:1 group (*P* = 0.0320). The results showed that a proper increase of L. acidophilus can promote the growth of other strains in the starter. Meanwhile, according to the result of the AI-2 assay, the fluorescence value in the mix culture condition was also higher than in the monoculture groups (Fig. 1C and D). Besides the OD_600_, the abundance of different strains was quantified using qPCR methods. The results showed that the number of strains was significantly different from the ratio of the strains (Fig. 2C). When fermented for 6 h, the relative numbers of L. bulgaricus, S. thermophilus, and L. acidophilus were 0.35. At the 24 h culture time, the related ratio of L. acidophilus reached 0.4. Meanwhile, in the 1:1:2 group, the number of L. bulgaricus also increased as the fermentation time increased (Fig. 2E to G).

### The tolerance of the LAB strains in the QS-related stimulated GIT condition.

In the QS-related stimulated GIT condition, the resistance ability of the mixed strain was increased in the gastrointestinal fluids environment when L. acidophilus was present. The fluorene density of AI-2 in all the groups was increased with the culture time from 0 to 24 h, while the AI-2 did not change after 24 h. The relative fluorescence intensity of the 1:1:1 group in gastrointestinal fluids at 24 h was significantly higher than L. acidophilus groups, which was close to 5500 AU (*P* = 0.0032). Meanwhile, compared with the 1:1 group, the fluorene density value was significantly higher in the 1:1:1 group with L. acidophilus (*P* = 0.0023), which meant the coculture condition would enhance the release of AI-2 molecule when cultured with L. acidophilus (Fig. 2A). However, the AI-2 activity between the two treated groups was not significantly different (*P* = 0.0786) at 24h (Fig. 2A and B). After the gastrointestinal fluid treatment, the number of the strains in the coculture condition was much higher than other groups, especially the 1:1:1 group, and the relative number of L. acidophilus was much higher than the other two strains (Fig. 2C and E to G). Furthermore, the *LuxS* gene involved in the synthesis of AI-2 was verified by qPCR, and the results showed that the *LuxS* genes of L. acidophilus were upregulated at 18 h compared to 12 h of cultivation and the expression level was twice as much as it was at 12 h (Fig. 2D).

### Metabolomics analysis.

**FIG 3:**
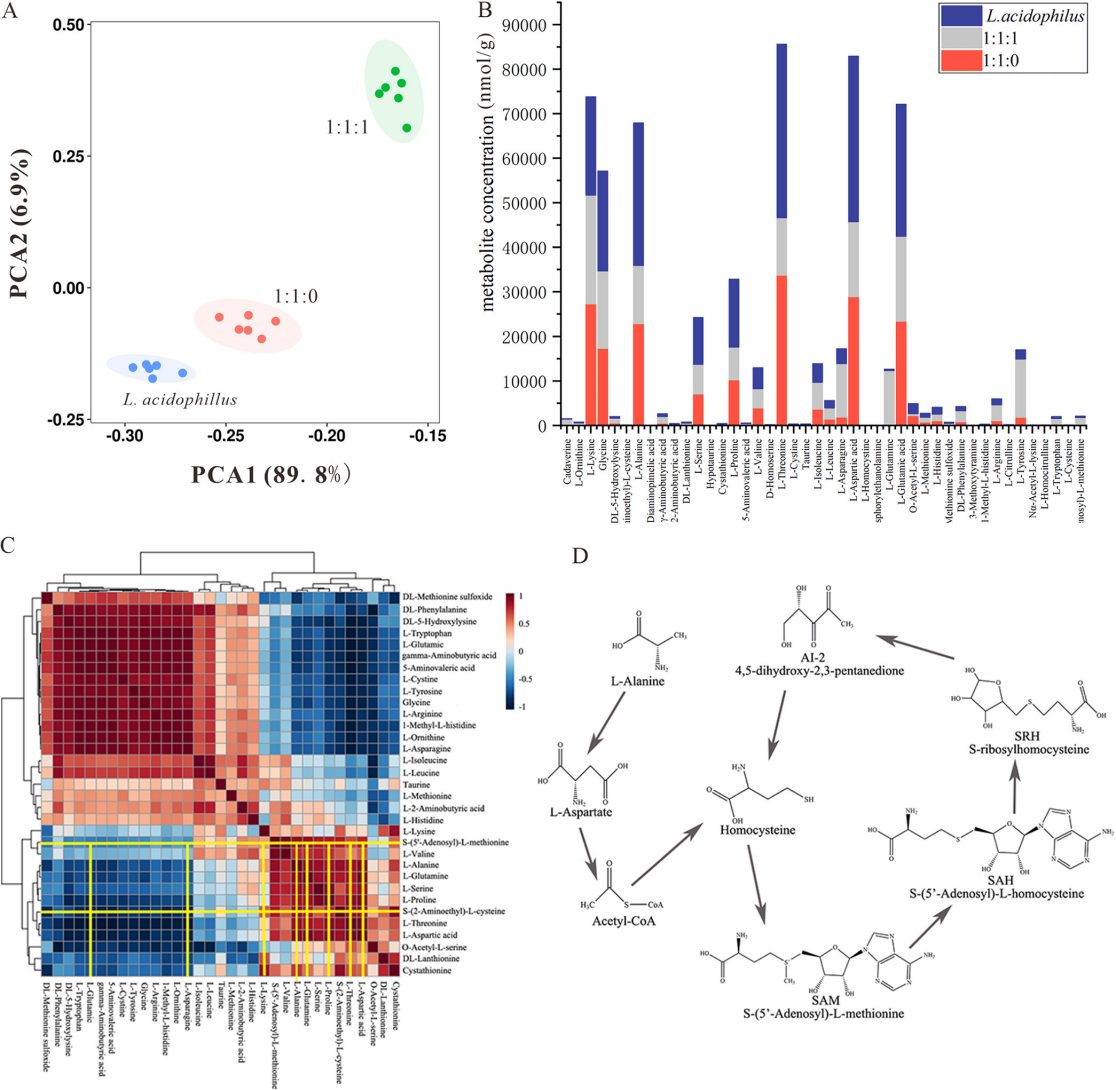
Amino metabolites analysis of culture media under the coculture conditions. (A) The PCA analysis of metabolites in the different mix-culture ratios. (B) The concentration of different metabolites in the different mix-culture groups. (C) The correlation analysis of the differential metabolites. (D) The synthesis pathway of SAM and SAH in the amino acid metabolites pathway.

In the multivariate statistical analysis of the metabolomics profiles, we investigated the changes of amino metabolites of different strains. The principal component analysis (PCA) score chart showed the apparent metabolite spectrum of L. acidophilus, L. bulgaricus:S. thermophilus 1:1, L. bulgaricus:S. thermophilus:L. acidophilus 1:1:1, which explained the metabolic differences of the samples (Fig. 3A). Amino acids, such as l-alanine and l-proline, were favorable to the release of S-(5′-Adenosyl)-l-methionine (SAM) and S-(5′-Adenosyl)-l-homocysteine (SAH). However, some amino acids were negatively correlated with the release of S-(5′-Adenosyl)-l-methionine (SAM) and S-(5′-Adenosyl)-l-homocysteine (SAH), such as l-glutamic acid and l-asparagine. In addition, stacked histogram analysis also showed the concentration of detected metabolites by the multivariate statistical analysis (Fig. 3B). Meanwhile, the heat map of the correlation between different metabolites showed whether the relationship between each metabolite was positive or negative (Fig. 3C). The metabolites pathway related to the production of AI-2 were given. From the metabolomics analysis, the results showed that l-alanine promoted the synthesis of homocysteine and further synthesized SAM and SAH (Fig. 3D).

**FIG 4:**
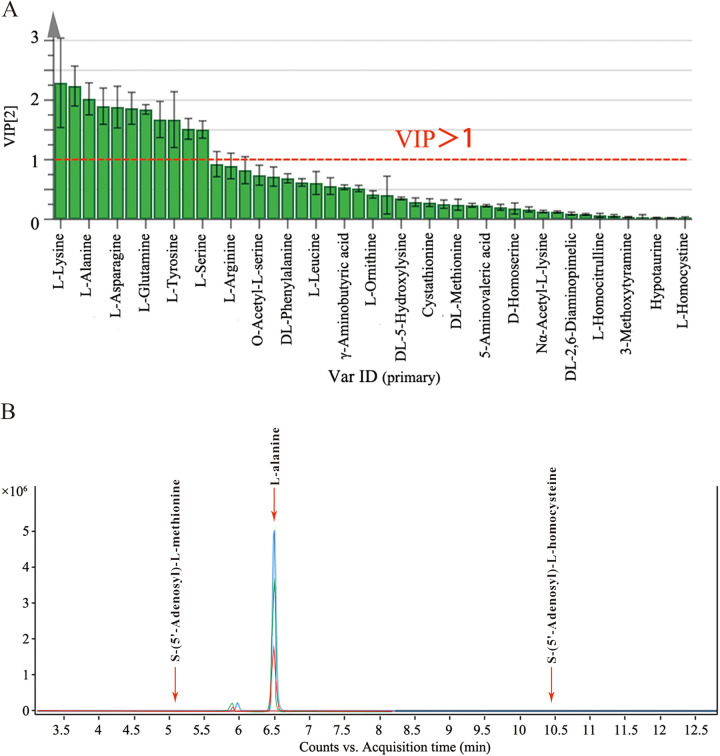
VIP analysis and chromatogram of differential metabolites. (A) The VIP analysis of metabolites; (B) the chromatograms of SAM, l-alanine, and SAH in different fermented groups. green represents the L. acidophilus fermented group; red represents the 1:1:0 fermented group; blue represents the 1:1:1 fermented group.

The variable importance of projection (VIP; obtained by partial least-squares discrimination analysis [PLS-DA]) value combined with the *P* value was used as the screening criterion to obtain significantly different metabolites (Fig. 4A). If the metabolite meets the parameter of VIP > 1 and *P* < 0.05, then it was proved to be a different metabolite. These differential metabolites are given in Table S1 and compared with the concentrations of the other 5 metabolites, and l-alanine was a significantly different metabolite. Through KEGG metabolic pathway analysis, the results also showed that l-alanine played an essential role in the synthesis of SAH and SAM (Fig. 3D).

### Amino acid-derived l-Alanine on the AI-2 release and growth characteristics of the strains in the coculture condition.

**FIG 5:**
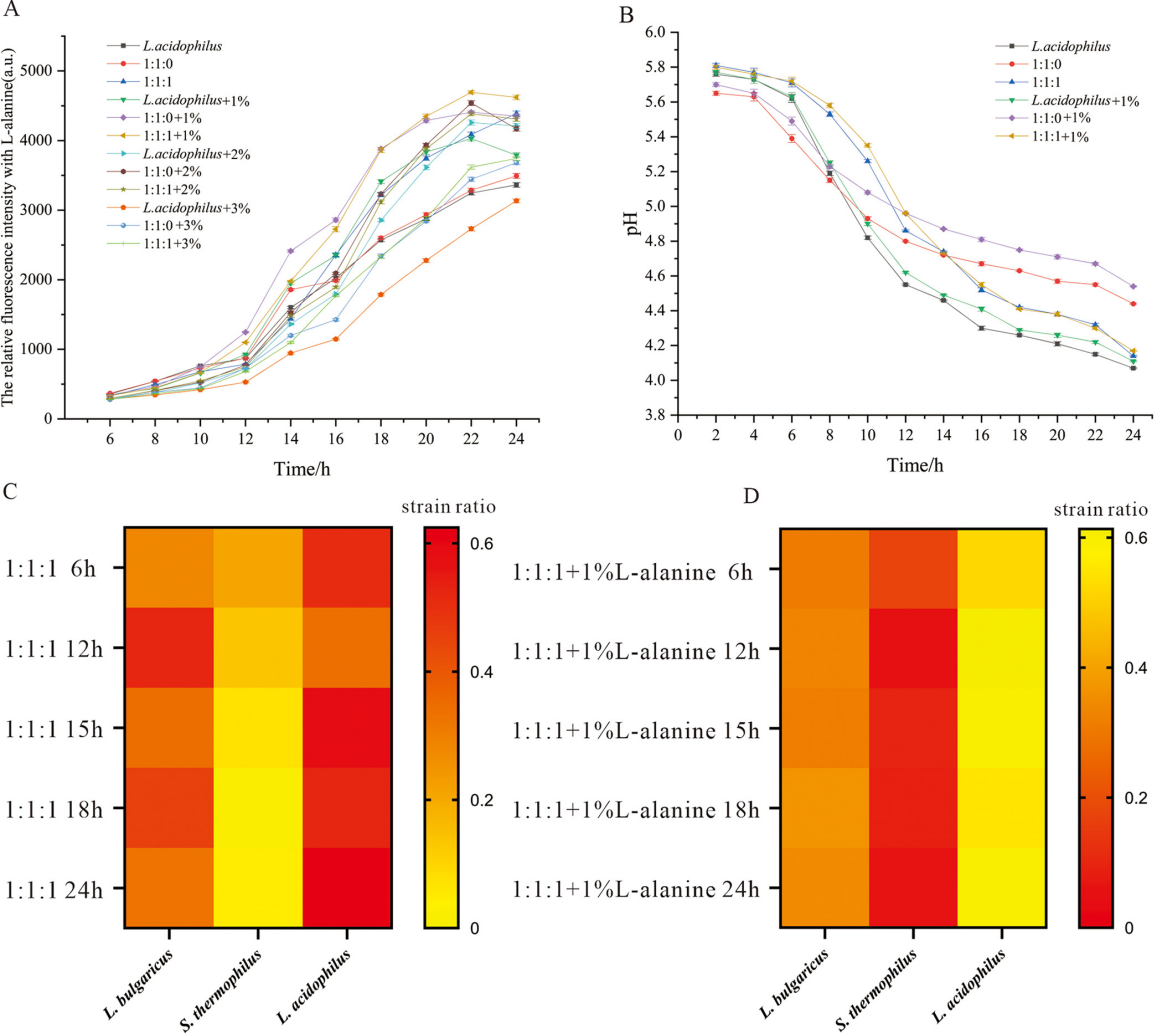
The relative fluorescence intensity and bacterial growth of coculture condition pretreated with l-alanine. (A) The relative fluorescence intensity of coculture media when pretreated with l-alanine; (B) the pH value of coculture media when pretreated with l-alanine. (C) The bacterial growth ratio in the coculture condition pretreated. (D) The bacterial growth ratio in the coculture condition pretreated with l-alanine.

In this study, strain V. harveyi BB170 was used to show the effect of amino acid-derived l-alanine on the release of AI-2 molecules in the coculture condition. The results showed that, when cultured with a 1% concentration of l-alanine, the secretion of AI-2 molecules was significantly increased compared to the group without l-alanine. After adding 1% l-alanine to the 1:1:1 group, the relative fluorescence intensity at 22 h was 4500. For the 1:1:1 group, it was 3500. After 22 h of culture, the release of AI-2 molecules tended to be stable (Fig. 5A). The addition of 1% l-alanine could significantly promote (*P* = 0.0034) the growth of L. acidophilus (24 h), which could be seen from the relative number of the strains in the heatmap illustration (Fig. 5C and D). However, the bacterial acid production did not increase after adding 1% l-alanine (Fig. 5B), and this was similar to the cell density at OD_600_ (Fig. S1).

**FIG 6:**
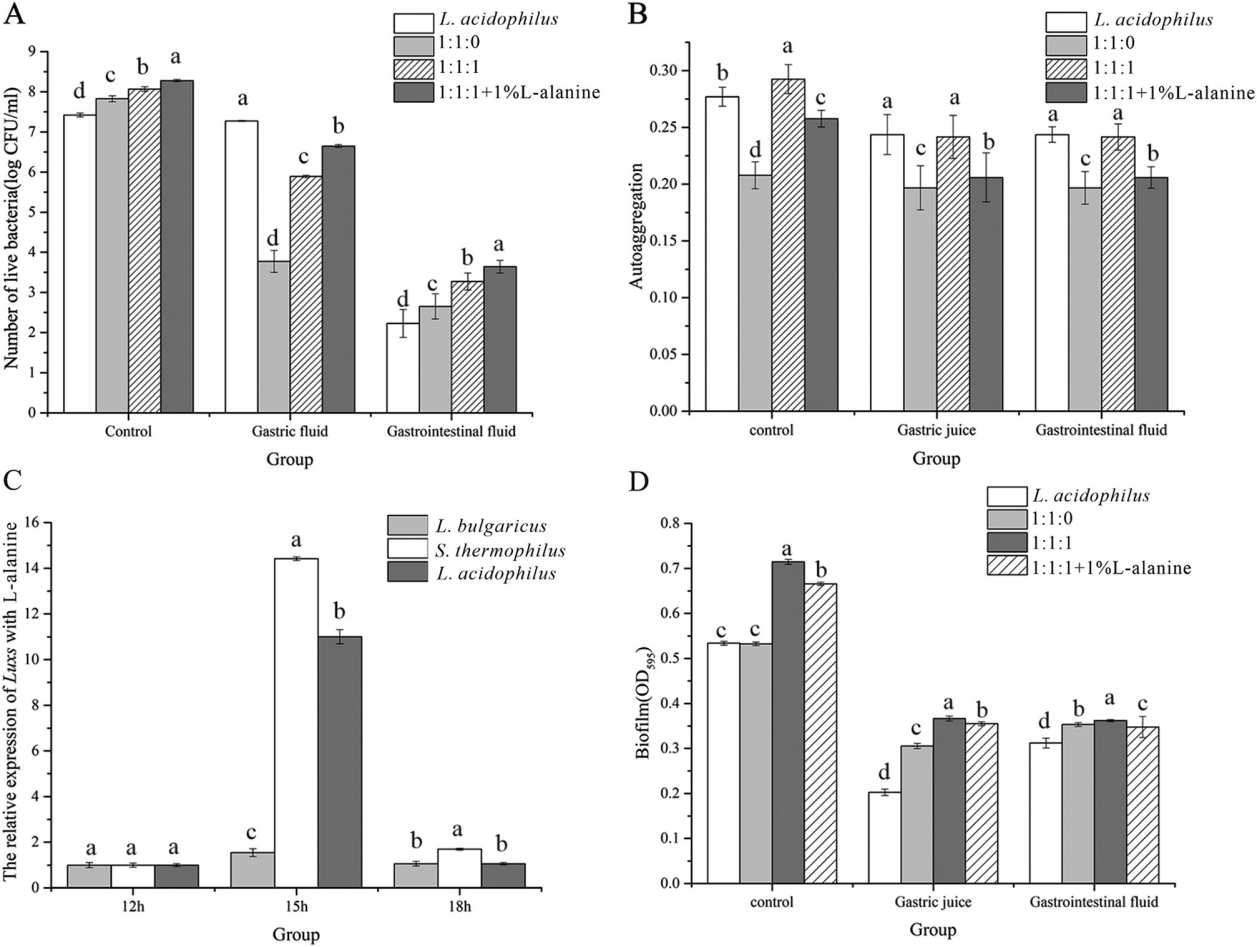
Various physiological indexes of bacteria in the coculture condition with l-alanine (1%). (A) The number of live bacteria in the gastrointestinal fluid under coculture conditions with 1% l-alanine. (B) The autoaggregation rate in the gastrointestinal fluid under coculture conditions with 1% l-alanine. (C) The relative expression of *LuxS* under coculture conditions with 1% l-alanine. (D) The biofilm formation in the gastrointestinal fluid under coculture conditions with 1% l-alanine. Lowercase letters indicate differences within groups, *P* <0.05 was used to indicate significant differences.

Moreover, amino acid-derived l-alanine (1%) can effectively promote the tolerance of mixed bacteria in the gastrointestinal juice condition. After gastric juice culture, compared with the 1:1:1 group, the number of viable bacteria in the 1:1:1 with 1% l-alanine group was 0.8 CFU/mL higher (Fig. 6A). In the simulated GIT environment, l-alanine significantly affected the viability, adhesion, and expression of the *LuxS* of the cocultured bacteria, especially in L. acidophilus (*P* = 0.02). However, the changes of the auto-aggregation were not significant, and there was no positive correlation between surface properties of lactic acid bacteria and adhesion ability (Fig. 6B). In addition, l-alanine (1%) could effectively promote the expression of *LuxS* after 15 h of culture. However, the expression of *LuxS* in the 12 h and 18 h groups were significantly lower than that in the 15 h group (Fig. 6C). The results of the adhesion ability of the L. acidophilus upon 1% l-alanine treatment showed that the mixed-culture condition with the 1% l-alanine could effectively enhance bacterial adhesion in the gastrointestinal environment (Fig. S2).

### Biofilm formation and liability in the coculture condition.

**FIG 7:**
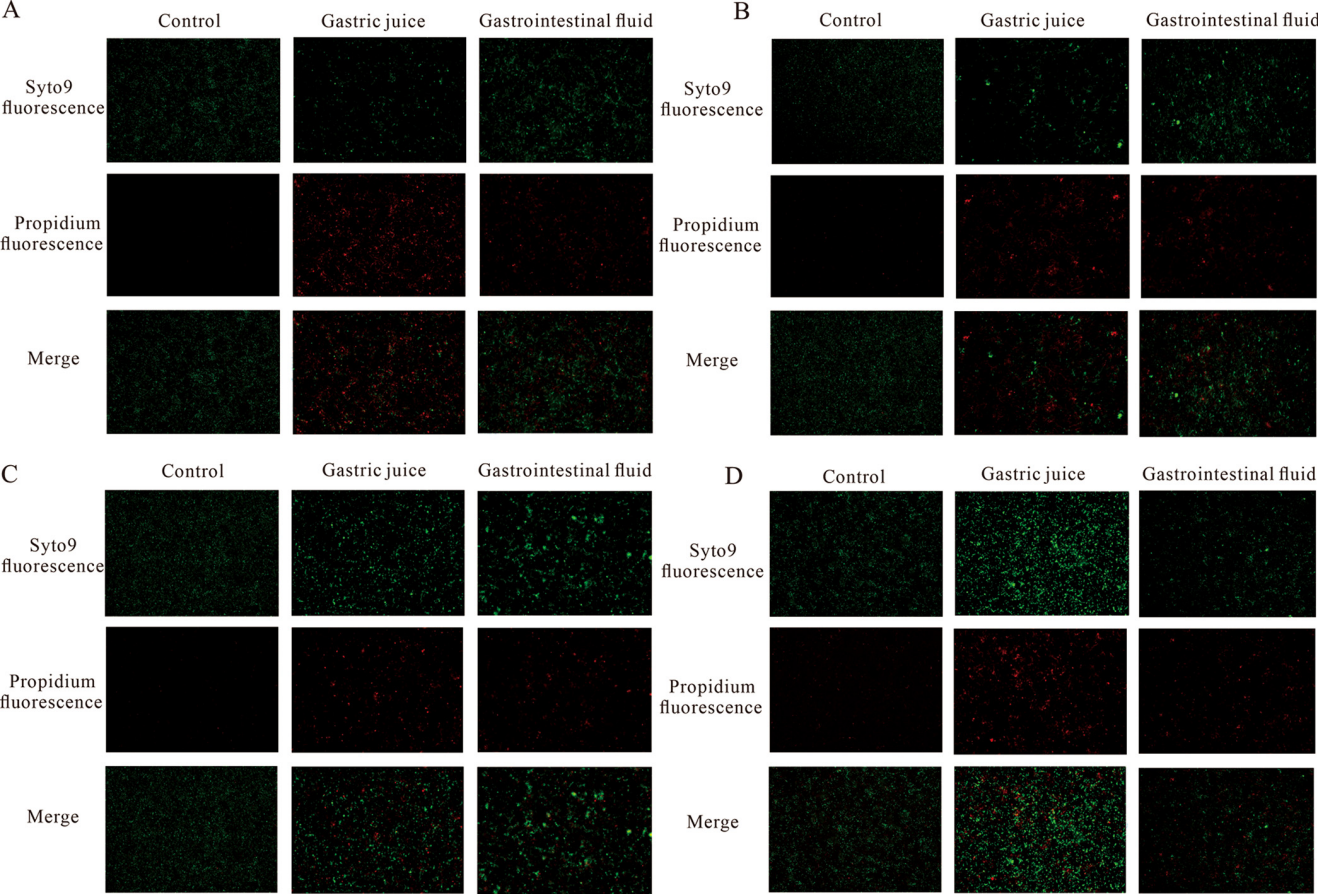
The viability of the bacteria represents with *in situ* fluorescent biofilm staining. (A) The survival of bacteria in L. acidophilus. (B) The survival of bacteria in 1:1:0. (C) The survival of bacteria in 1:1:1; (D) the survival of bacteria in 1:1:1 with 1% l-alanine.

The results showed that the biofilm of L. acidophilus was significantly reduced after gastrointestinal fluid culture (*P* = 0.0046). Compared to L. acidophilus monoculture, the 1:1:1 coculture condition significantly promoted biofilm production (*P* = 0.0377). It was also affected by gastric juice and intestinal juice, but the coculture condition could weaken the inhibitory effect of gastric and intestinal fluids on biofilms (Fig. 6D). The Live/Dead BacLight cell viability test is an effective method for the detection of the viability and biofilm changes of the bacteria in different conditions. As shown in Fig. 7, LAB in the coculture condition could enhance the cell liability more than the monoculture condition, and the number of alive bacteria in the 1:1:1 group after stimulated GIT environment treatment was much higher. Meanwhile, the fluorescence intensity was also slightly enhanced when cocultured with 1% l-alanine.

## DISCUSSION

As a common probiotic, *Lactobacillus* is beneficial to the regulation of intestinal flora and enhances the interaction of gut microbiota with the host. Meanwhile, the use of the *Lactobacillus* strains in functional foods for health promotion purposes has increased significantly (21). While numerous researchers have focused on the ability of various probiotic strains to influence host immune responses beneficially, metabolic processes, and neuroendocrine pathways (22), further studies related to the interaction of different probiotic strains with the gut microbes and the host intestinal cells still needs to be considered.

In this study, when L. bulgaricus, S. thermophilus, and L. acidophilus were cocultured at the ratio of 1:1:1, the bacterial density was increased significantly, and the pH value of the cocultured medium was also more suitable for the growth of the *Lactobacillus* strains, especially L. acidophilus. Previous studies have found that when L. acidophilus is cocultured with *Bifidobacteria*, the environment becomes acidic due to its acid-producing ability, which promotes the growth of L. acidophilus. At the same time, the cocultured conditions enhanced the liability of the strains (23). There is a particular way of communication between strains under cocultured conditions. Quorum sensing is a way of signal transmission between bacteria (24). In this study, the production of QS-related signal molecules in the mixed culture medium was much higher than of the separately cultured, and the participation of L. acidophilus also promoted the generation of signal molecules. L. acidophilus has been reported to promote its intestinal adhesion ability and adaptation by AI-2 quorum sensing (25). Meanwhile, research also found that when L. plantarum DC400 cocultured with L. sanfranciscensis DPPMA174 or L. rossiae A7, the relative fluorescence intensity in the mixed strain groups was significantly higher than that of the single cultured. The expression of the *LuxS* gene was 2.5 and 3.5 times that of a single-cultured group (26). This is consistent with our present finding that L. acidophilus could promote the production of QS-related signal molecules in the mixed-culture condition. During the growth and reproduction of bacteria, signal molecules encoded by specific genes can be released. The AI-2 signal molecules produced by the *LuxS* enzyme-catalyzed by *LuxS* gene encoding between Gram-positive bacteria carry out signal transmission (27). Our result also showed that the content of the *LuxS* gene in L. acidophilus was much higher than that of L. bulgaricus and S. thermophilus during the culture process. Meanwhile, although the coculture condition did not promote bacterial proliferation, the coculture condition could promote the growth of L. acidophilus. This is also the reason the *LuxS* gene was much higher than other groups. Due to the limitation of growth space and nutrients *in vitro*, the growth curves measured in the flask by the experimental groups are not much different, but the growth of each strain in the coculture condition was quite different.

Bacteria are known to release many small molecules, including siderophores, secondary metabolites, and metabolic end products (28). There are many metabolites containing amino groups in organisms, including protein amino acids, nonprotein amino acids, modified amino acids, and these amino metabolites cover multiple metabolic pathways in the organism. In the methionine metabolic pathway, methionine and ATP are activated by methionine adenosyltransferase, and methionine derivatives, such as SAM is the methyl donor for the synthesis of AI-2 (29). In the metabolite analysis performed using UPLC-MS/MS, l-alanine was screened as the significant amino acid-derived quorum sensing molecule through multivariate statistical analysis and the variable importance of projection analysis. In the KEGG metabolic pathway analysis, l-alanine could promote the synthesis of SAM and SAH, and the addition of 1% l-alanine could enhance the growth of LAB, especially the growth of L. acidophilus, and the expression of the *LuxS* gene-level in the cocultured model.

In addition to the critical interaction between LAB, another important feature of the LAB strain is their survival ability in the gastrointestinal tract (30). In 1989, Fuller pointed out that probiotics must be living microorganisms and have a beneficial impact on the host (31). Our result showed that the coculture condition effectively protects the LAB in the gastrointestinal environment, and amino acid-derived QS molecule l-alanine can work both as the QS regulator in the synthesis of AI-2 and as the enhancer, increasing the GIT tolerance of the *Lactobacillus* strains in the cocultured model. Furthermore, in the coculture condition, the viability, self-aggregation rate, adhesion to cells, and biofilm formation of the bacteria also had a relationship with each other during this special situation. Furthermore, for the bacterial adhesion, l-alanine was a positive stimulator in the cell adhesion model, while there was no positive correlation between surface properties of lactic acid bacteria and adhesion ability and the biofilm formation in the coculture of 1% l-alanine.

Previous studies found that cocultured L. plantarum and P. acidilactici VTCC 10800 or S. cerevisiae Y11-43 significantly improved the biofilm formation ability (32, 33). In addition, biofilm detection results under monoculture and coculture conditions also confirmed this conclusion. The formation of biofilm could increase the liability of the cell, and the coculture condition of LAB will provide a platform for the communication of the bacteria to interact with each other.

In summary, it is shown that a cocultured condition is a practical approach for the interaction of the LAB in the fermentation model in this research. Meanwhile, the special and natural fermentation process can promote the release of amino acid-derived quorum-sensing molecules that can effectively enhance the communication and liability of LAB in the simulated gastrointestinal environment by increasing the adhesion properties of the strain. The findings of this study confirmed the QS of the LAB in the coculture condition *in vitro* and provide a clue for using the amino acid-derived quorum sensing molecule in the complex fermented dairy industry.

## MATERIALS AND METHODS

### Strains and culture conditions.

Lactobacillus acidophilus CICC6074 was obtained from the China General Microbiological Culture Collection Center (Beijing, People’s Republic of China). Streptococcus thermophilus ABT-T and Lactobacillus delbrueckii subsp. *bulgaricus* BNCC336436 were stored in our laboratory. In the experiment, all cells were cultured in MRS medium at 37°C for 12 h before use.

### Growth characteristics of the strains in coculture conditions.

L. bulgaricus, S. thermophilus, and L. acidophilus were investigated for their growth characteristics under mixed-culture conditions, their OD_600_ values were adjusted to 1.0 ± 0.1 to ensure the same initial strain density, and they were cultured at 37°C in MRS medium under static conditions with different initial inoculation ratios (1:1:0, 1:1:1, 1:1:2, 1:1:3). The growth rate (OD at 600 nm) and the pH value of the strains under the mixed-culture conditions were measured at every 2 h, respectively, during the 24 h culture time.

Quantitative analysis of the account of the L. bulgaricus, S. thermophilus, and L. acidophilus in the cocultured media at different fermentation times was detected by the quantitative real-time PCR (qPCR) with the gene-specific primer design with the Primer Premier 5.0 software. The 16S rRNA gene-specific primers used in the qPCR analysis were as follows: L. bulgaricus, forward (5′-GCAAGTCGAGCGAGCTGAATT-3′), reverse (5′-GCACCTGTTTCCAAGTGGTATCC-3′); S. thermophilus, forward (5′-ATACCGCATAACAATGGATGACACA-3′), Reverse (5′-CTCTCAGGTCGGCTATGTATCGT-3′); and L. acidophilus, forward (5′-GCAAGTCGAGCGAGCTGAAC-3′), reverse (5′-GCACCTGTTTCCAAGTGGTATCC-3′).

### Gastrointestinal tolerance analysis.

The tolerance ability of the L. bulgaricus, S. thermophilus, and L. acidophilus under mixed-culture conditions was investigated according to the method of Ranadheera et al. (34) with some modifications. The mixed-strain culture (18 h, 4 mL) from the different inoculation ratios (1:1:0, 1:1:1) was harvested by centrifugation (6000 × *g*, 10 min). After that, the collected mixed bacteria were resuspended in 4 mL artificial gastric juice (pH 2.0) and incubated for another 3 h at 37°C. After treatment with artificial gastric juice, 5g NaCl was dissolved in 1 liter ultrapure water, and the pH was adjusted to 2.0. After sterilization, 3 g pepsin was added and filtered with 0.22 μL filter membrane), bacteria were collected by centrifugation (6000 × *g*, 10 min) and resuspended in simulated intestinal fluids (dissolve 5 g NaCl and 3 g bovine bile salt in 1L ultrapure water) and adjusted pH to 8.0. After sterilization, 1 g trypsin was added and filtered with 0.22 μL filter membrane) for another 4 h. The CFU of the existing viable bacteria was determined before/after *in vitro* gastrointestinal digestion and the survival rates were calculated with three independent tests.

### AI-2 bioassay detection.

Autoinduction of luminescence in the marine bacteria V. fischeri and V. harveyi was described in the early 1970s (35). In this study, the AI-2 reporting strain V. harveyi BB170 was adopted for the detection of signaling molecules, and the AI-2 bioassay was performed according to the method as previously described (36). Cell-free culture fluid (CF) was prepared as follows. After centrifuging the test strain at 4600 × *g* for 10 min, the resulting supernatant was further filtered through a 0.22 μm filter to obtain the CF sample. The reporter strain V. harveyi BB170 was diluted 1:5000 in fresh AB medium, and then 99 mL of AB culture was mixed with 1 mL of the CF sample fluid and incubated at 30°C. The fluorescence intensity was detected every 1 h with the black 96-well microplate reader, the excitation and emission wavelengths were 485 nm and 538 nm, respectively. Experiments were performed in triplicate and repeated at least three times.

### Quantitative reverse transcription-PCR for the detection of the *LuxS* signaling molecular gene.

The strains L. acidophilus, L. bulgaricus, and S. thermophilus (1:1:1) were inoculated in the MRS medium at 37°C for 18h. The RNA samples of the cells were then collected using a Magen kit (Magen Biotech Co. Ltd., Guangzhou, China). After that, the extracted RNA was reverse transcribed with the kit (TranScript All-in-One First-Strand cDNA Synthesis SuperMix for qPCR, TransGen Biotech, Beijing, China) for qPCR analysis with the internal reference gene was *dp3d* (DNA polymerase III, delta subunit). Gene-specific primers were forward (5′-GAATGTGGGCGTTAAGCAAACC-3′) and reverse (5′-TGCACGTTCCTCATCACTATCG-3′).

Gene-specific primers of the *LuxS* in different strains were as follows. L. bulgaricus, forward (5′-CACACCATTGAACACCTCCTG-3′), reverse (5′-TGTCGTCTCTGATTGCCTTGA-3′); S. thermophilus, forward (5′-TTTGGTTGCCGTACAGGTTTCC-3′), reverse (5′-GCTGAATGAAGGCTGTGATCCT-3′); and L. acidophilus, forward (5′-CCTACCGGCGGATTGCATACTA-3′), reverse (5′-ATCCTGTTCGGCAACCAAATGG-3′).

qPCR analysis was carried out in triplicate with the TransStart Tip Green qPCR SuperMix kit (TransGen Biotech, Beijing, China), and the number of cDNAs were normalized to the abundance of *dp3d* by the 2^-△△CT^ method. The relative quantitative method was used for *LuxS* signaling molecule gene analysis.

### Metabolomics analysis.

The harvested mixed-cultured LAB (18 h) were centrifuged (4600 × *g*, 5 min) and washed twice with double-distilled water. The supernatant was then removed (4600 × *g*, 5 min), and the bacterial samples were lyophilized by vacuum-freeze drying. Bacterial samples (10 mg) were resuspended in 1.5 mL precooled methanol and subjected to three freeze-thaw cycles before sonication in an ice bath for 15 min (cycles: 1 min pulse followed by 1 min pause). An aliquot (10 μL) was vortex-mixed with 10 μL of N-Ethymaleimide (NEM) solution (20 mM) in phosphate buffer (0.1 M, pH 7.0) containing 10 mM ascorbic acid, 10 mM EDTA, and 7% DMSO for 1 min. 10 μL tBBT solution (0.23 M in DMSO) was added followed by the addition of 87.5 μL borate buffer (0.2 M, pH 8.8) containing 20 mM Tris (2-carboxyethyl) phosphine (TCEP) and 1 mM ascorbic acid. After vortex-mixing and standing for 2 min, 33 μL 5-Aminoquinoline (5-AIQ) solution was then added and incubated at 55°C for 10 min. The mixture was cooled down to the ambient temperature and added with 2 μL formic acid followed with the solution was filtered by a 0.22 μm membrane filter before ultra-performance liquid chromatography-tandem mass spectrometry (UPLC-MS/MS) analysis.

The UPLC-MS/MS consisted of an Agilent 1290 UPLC coupled to an Agilent 6470 triple quadrupole mass spectrometer equipped with an electrospray ionization (ESI) source (Agilent Technologies, USA). The 5-AIQ-tagged samples (1 μL) were individually injected on a UPLC column (Agilent ZORBAX RRHD Eclipse XDB C18 column, 2.1 × 100 mm, 1.8 μm particles) with its temperature set to 50°C. Water and methanol containing 0.1% (vol/vol) formic acid were used as two mobile phases A and B, respectively, with a flow rate of 0.5 mL/min. An optimized gradient elution scheme was employed as 1% B (0 to 2 min), 1 to 3.8% B (2 to 4 min), 3.8 to 14% B (4 to 7.3 min), 14 to 22% B (7.3 to 10.7 min), 22 to 24% B (10.7 to 14.7 min), 24 to 30% B (14.7 to 16 min), 30 to 60% B (16 to 16.3 min), 60 to 70% B (16.3 to 17.3 min), 70 to 95% B (17.3 to 17.31 min), and 95% B (17.31 to 20 min). Electrospray ionization was performed in the positive ion mode using N^2^ at a pressure of 50 lb/in^2^ for the nebulizer with a flow of 10 L/min and a temperature of 315°C, respectively. The sheath gas temperature was 350°C with a flow rate of 10 L/min. The capillary was set at 4000 V. Multiple reaction monitoring (MRM) has been used for the quantification of screening fragment ions.

The metabolism experiments were repeated six times and the quality control samples (QC samples) were analyzed in continuous random order. Peak determination and peak area integration were performed with MassHunter Workstation software (Agilent, Version B.08.00). Standard curves were constructed by least-squares linear regression analysis using the peak area ratio of derivatized individual standard against the nominal concentration of the calibrator. Quantification of samples was performed identically. OriginPro 9.1 and SIMCA 14.1 were used for multivariate statistical calculations and plotting.

### Influence of characteristic metabolites on the QS, growth, and tolerance characteristics of the strains in coculture conditions.

The characteristic metabolite was added to the coculture system (L. bulgaricus:S. thermophilus:L. acidophilus 1:1:0 and 1:1:1) in different proportions (1%, 2%, and 3%). The AI-2 and *LuxS* were detected according to the method as previously described. Meanwhile, the growth and tolerance characteristics were performed according to the method as previously described with 1% characteristic metabolites in the coculture system (L. bulgaricus:S. thermophilus:L. acidophilus 1:1:0 and 1:1:1).

The Live/Dead BacLight cell viability test kit can reliably and quantitatively quickly distinguish live bacteria from dead bacteria. Bacteria with intact cell membrane showed green fluorescence, while bacteria with damaged cell membrane showed red fluorescence. In this study, cell viability was detected after the stimulated gastrointestinal condition treatment. Mix equal volumes of Syto 9 and propidium were added in a centrifuge tube and then 3 μL of dye mixture was added to the bacteria suspension and incubated for 30 min in the dark. The image of the Live/Dead bacteria was observed using an inverted fluorescence microscope.

### Aggregation assay.

The aggregation assay was according to the method of Cao et al. (37) with some modifications. Different mixed-culture groups (1:1:0 and 1:1:1) were grown in MRS medium for 18 h at 37°C, centrifuged at 4600 × *g* for 10 min, washed twice with PBS, and adjusted the value of OD_600_ to 1.0 ± 0.05, which is A_0_. The bacterial suspension (4 mL) mix was incubated at room temperature for 4 h, and the absorbance was measured at 600 nm (A_4_). The self-aggregation rate (%) was calculated as (1-A_4_/A_0_) × 100, where A_4_ and A_0_ represented the absorbance at 4 h and 0 h, respectively. The aggregation assay of the bacteria with the gastrointestinal fluid treatment was calculated with the same experimental procedure above.

### Adhesion assay *in vitro*.

The adhesion assay was carried out with the Caco-2 cell model. After the gastrointestinal treatment, the LAB samples were stained with the 6-carboxyfluorescein diacetate (CFDA, 10 μM, Sigma, Darmstadt, Germany). When the Caco-2 cells covered 80% of the area of the petri dish bottom, the stained bacteria were cocultured with the Caco-2 cells for 2 h at 37°C. Finally, fluorescence intensity (excitation wavelength 485 nm; emission wavelength 538 nm) was measured using Tecan Infinite M200 Pro (Shanghai, China), and the adhesion rate was expressed as the percentage of fluorescence after binding to Caco-2 cells relative to the bacterial suspension added to the same well.

### Biofilm formation detection.

The biofilm formation of the strain in the coculture condition was detected according to the method of Rieu et al. (38). L. bulgaricus, S. thermophilus, and L. acidophilus (1 × 10^8^ CFU/mL) were added to a 24-well plate at different ratios and cultured at 30°C for 24 h. Bacteria that were not bound to the bottom of the well plate were washed with 150 mM NaCl solution. For the simulated gastrointestinal environment analysis, the same amount of the gastric juice or intestinal juice was added to the well plate and incubated for 2 h, and then wash twice with NaCl solution (150 mM). For the staining procedure, the control group and the treatment group were stained with 0.05% crystal violet for 45 min. After washing the unbounded crystal violet, 96% ethanol was added to dissolve the crystal violet for another 45 min, and the absorbance was measured with a microplate reader at 595 nm.

### Statistics.

Data were analyzed using the one-way analysis of variance (ANOVA) and independent-samples test by SAS 9.1 software with *P* < 0.05 considered significant. Multi-Experiment Viewer (MeV) software was used to quantify the different strains in the coculture condition by the microarray heatmap, which combined with hierarchical cluster analysis (HCA). The unsupervised model (PCA) and partial least-squares discrimination analysis (PLS-DA) model were established to evaluate the impact of metabolites by using SIMCA-P software.
