# Supplementary material for: Amino Acid-Derived Quorum Sensing Molecule Alanine on the Gastrointestinal Tract Tolerance of the Lactobacillus Strains in the Cocultured Fermentation Model
Source: Microbiol Spectr. 2022 Mar 3;10(2):e00832-21. doi: 10.1128/spectrum.00832-21 (PMC9045186; doi:10.1128/spectrum.00832-21)
Supplement: SUPPLEMENTAL FILE 1 — Supplemental material. Download spectrum.00832-21-s0001.pdf, PDF file, 0.2 MB [file spectrum.00832-21-s0001.pdf]

Table S1 Concentration of each metabolite in different culture group

Amino acid	concentration (nmol/g)		
	L. acidophilus	1: 1: 0	1: 1: 1
Cadaverine	43.40±7.24[b]	1401.61±31.29[a]	32.37±3.16[b]
L-Ornithine	64.56±14.14[c]	523.28±37.21[a]	147.22±28.51[b]
L-Lysine	27327.13±1933.11[a]	24823.18±1408.38[b]	21535.35±834.07[c]
Glycine	17341.49±622.62[b]	17242.74±817.06[b]	22265.93±730.11[a]
DL-5-Hydroxylysine	567.40±37.27[b]	1065.54±60.09[a]	362.46±14.96[c]
S-(2-Aminoethyl)-L-cysteine	50.10±4.90[a]	8.18±0.45[c]	43.06±2.84[b]
**L-Alanine**	**22868.19±1128.31[b]**	**13380.85±1663.85[c]**	**31990.93±1016.93[a]**
DL-2,6-Diaminopimelic acid	15.95±1.74[b]	22.31±2.07[a]	23.77±1.00[a]
gamma-Aminobutyric acid	529.88±29.19[b]	1497.74±112.19[a]	565.01±23.60[b]
L-2-Aminobutyric acid	103.40±8.13[c]	181.92±14.15[a]	154.33±10.36[b]
DL-Lanthionine	300.40±17.83[a]	216.34±18.10[b]	231.72±9.20[b]
L-Serine	7057.00±217.65[b]	6674.26±437.10[b]	10457.96±396.63[a]
Hypotaurine	4.62±0.67[b]	7.58±0.71[a]	3.51±0.43[b]
Cystathionine	179.68±11.00[a]	103.14±10.64[b]	117.56±4.96[b]
**L-Proline**	**10239.57±469.12[b]**	**6968.29±1442.79[c]**	**15111.51±510.98[a]**
5-Aminovaleric acid	121.56±6.95[b]	286.88±17.07[a]	134.74±5.16[b]
L-Valine	3964.80±123.79[c]	4260.50±293.74[b]	4747.00±153.51[a]
D-Homoserine	14.09±1.18[b]	123.69±45.31[a]	13.18±0.80[b]
L-Threonine	33718.38±1200.23[b]	12578.31±1348.19[c]	38527.65±1067.69[a]
**L-Cystine**	**67.68±4.79[b]**	**169.47±26.97[a]**	**65.97±5.96[b]**
Taurine	111.97±7.22[ab]	117.25±5.86[a]	106.93±5.53[b]
L-Isoleucine	3677.13±193.17[c]	5996.84±494.00[a]	4102.54±161.78[b]
L-Leucine	1419.36±139.53[b]	2475.94±284.71[a]	1604.38±153.49[b]
L-Asparagine	1902.65±119.03[c]	12102.95±1128.16[a]	3245.87±191.57[b]
L-Aspartic acid	28969.62±1454.28[b]	17508.44±2267.50[c]	36825.54±809.10[a]
L-Homocystine	5.93±0.71[b]	7.63±2.00[a]	6.21±0.71[ab]
O-Phosphorylethanolamine	3.70±0.54[b]	5.71±0.79[a]	3.97±0.36[b]
L-Glutamine	202.05±15.00[c]	12033.97±854.09[a]	290.45±26.51[b]
**L-Glutamic acid**	**23433.34±1310.58[b]**	**19940.33±2853.68[c]**	**29461.44±493.36[a]**
O-Acetyl-L-serine	2240.55±222.68[a]	453.78±42.55[b]	2138.41±109.20[a]
L-Methionine	771.72±150.56[b]	1124.52±163.82[a]	926.73±78.26[b]
L-Histidine	1088.74±56.17[b]	1499.79±106.99[a]	1527.81±94.03[a]
DL-Methionine sulfoxide	181.33±6.82[c]	280.62±31.33[a]	236.33±47.19[b]
DL-Phenylalanine	871.17±78.44[b]	2424.04±188.69[a]	952.22±25.86[b]
3-Methoxytyramine	7.43±0.55[a]	5.24±0.67[b]	5.96±0.91[b]
1-Methyl-L-histidine	33.64±4.53[c]	173.68±18.78[a]	61.84±5.79[b]
L-Arginine	1130.43±47.74[c]	3636.40±359.39[a]	1340.98±91.46[b]
L-Citrulline	12.21±1.65[b]	147.91±18.97[a]	12.73±0.94[b]
L-Tyrosine	1863.94±983.16[b]	12835.35±3490.15[a]	1465.54±1039.62[b]
Nα-Acetyl-L-lysine	10.57±0.82[b]	71.86±4.60[a]	9.70±0.96[b]

L-Homocitrulline	21.03±1.65[b]	35.97±5.78[a]	23.10±1.58[b]
L-Tryptophan	342.60±16.33[b]	1245.49±82.75[a]	369.30±14.16[b]
L-Cysteine	13.71±1.08[b]	42.37±4.40[a]	12.90±2.22[b]
S-(5'-Adenosyl)-L-methionine	285.51±97.77[b]	1504.05±364.34[a]	227.15±26.43[b]

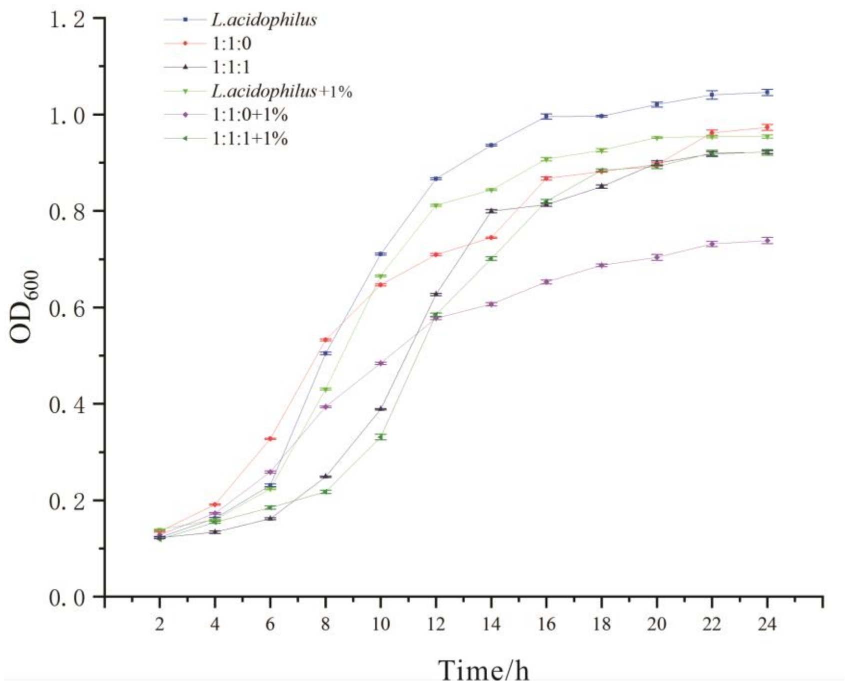

Figure S1. The OD600nm of co-culture media when pretreated with L-alanine.

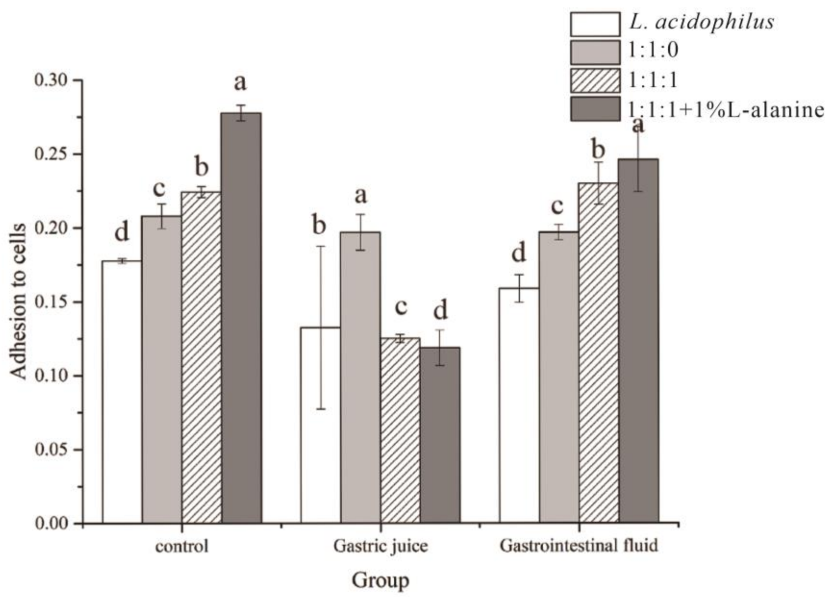

Figure S2. The adhesion to cells in the gastrointestinal fluid under co-culture conditions with 1% L-alanine.
